# A low cost, green method to synthesize GaN nanowires

**DOI:** 10.1038/srep17692

**Published:** 2015-12-08

**Authors:** Jun-Wei Zhao, Yue-Fei Zhang, Yong-He Li, Chao-hua Su, Xue-Mei Song, Hui Yan, Ru-Zhi Wang

**Affiliations:** 1 College of Materials Science and Engineering, Beijing University of Technology, Beijing 100124, China; 2 Inst. Microstruct. & Property Adv. Mat., Beijing University of Technology, Beijing 100124, China

## Abstract

The synthesis of gallium nitride nanowires (GaN NWs) by plasma enhanced chemical vapor deposition (PECVD) are successfully demonstrated in this work. The simple and green synthesis route is to introduce gallium oxide (Ga_2_O_3_) and nitrogen (N_2_) for the growth of nanowires. The prepared GaN nanowires have a single crystalline wurtzite structure, which the length of some nanowires is up to 20 μm, with a maximum diameter about 140 nm. The morphology and quantity of the nanowires can be modulated by the growth substrate and process parameters. In addition, the photoluminescence and field emission properties of the prepared GaN nanowires have been investigated, which were found to be largely affected by their structures. This work renders an environmentally benign strategy and a facile approach for controllable structures on nanodevice.

There is a strong ongoing interest in the use of semiconductor nanowires for active nanoscale electronic and photonic devices^1^,^2^,^3^. Among them GaN has attracted much attentions^4^,^5^,^6^. Due to its wide band gap, high melting temperature, carrier mobility, strong chemical stability and high electric breakdown field have demonstrated great potential in device applications^7^,^8^,^9^, including field-effect transistors (FET)^10^, light-emitting diodes (LEDs)^11^,^12^, ultraviolet photo-detectors and lasers applications^13^,^14^.

To date, the synthesis of GaN NWs have been achieved by various of technical routes, such as carbon-nanotube-confined reaction^15^, oxide-assisted method^16^, vapor-solid mechanism^17^ and vapor-liquid-solid mechanism^18^. Many types of source materials have been attempted such as trimethylgallium (TMG), metal gallium, gallium chloride (GaCl_3_) and gallium nitride (GaN), etc^9^,^19^,^20^,^21^, but most of them are either expensive or toxic. In addition, ammonia, which is corrosive and harmful, is always used as the reaction gas. In order to change the existing situations, recently, we developed an environmentally friendly method to synthesize GaN NWs by a customized plasma assisted hot filament CVD system^9^. In this technique, GaN powders were used as the Ga precursor, and a negative DC bias was used to decompose nontoxic N_2_ to create positive nitrogen ions. By this method, we successfully prepared GaN NWs with different morphologies, and exhibiting good photoluminescence properties. However, the crystallinity and productivity of GaN NWs are still unsatisfactory for this method. Therefore, we further improved this method. GaN powders were replaced by economic and widely accessible Ga_2_O_3_. Due to the existence of some oxides in this reaction system, the reducible carbon and hydrogen were introduced to guarantee that the generated nitride was not affected by the oxygen element in the reactants. Upon this improvement in a plasma enhanced chemical vapor deposition (PECVD) system, the GaN NWs have been successfully synthesized. Their structures can be significantly modulated by the process parameters. And the growth mechanism has been revealed. The photoluminescence and field emission properties of the prepared nanowires have been investigated. Our works suggests an environmentally friendly strategy and facile approach for one dimensional nano-structural nitride materials.

## Experiments and Mearsurements

In this work, gallium oxide (Ga_2_O_3_) and ionized nitrogen were used as Ga and N precursors, respectively. H_2_ and carbon were introduced to create an oxygen-free environment and reduce the degree of vacuum level required for the reaction. The growth procedure is as following: First, the substrate was chemically cleaned by toluene, acetone, ethanol and de-ionized water in an ultrasonic bath for 15 min in each solvent sequentially; after that, the other parameters were set according to the experimental requirements.

**Figure 1:**
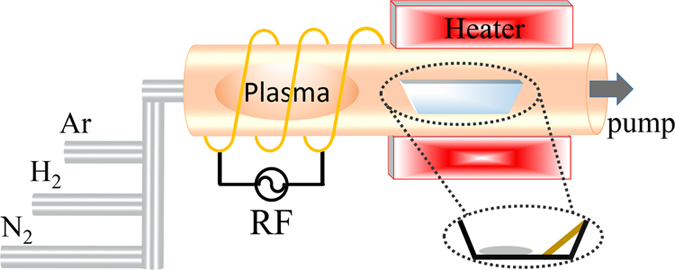
Schematic of home-developed PECVD system.

Then sputter coater (SBC-12) was introduced to deposit an Au film on all of the substrates for 10s. After that, about 0.2 g Ga_2_O_3_ (purity 99.999%) mixed with carbon (purity 99.9%) (Mole ratio 1:6) powders were placed in a small Al_2_O_3_ boat. The boat was placed at the highest temperature zone of a horizontal quartz tube, with the substrate located at the downstream end of the boat. The distance between them was about 5 mm, as depicted in Fig. 1.

The furnace was heated to 900 °C within 70 min in Ar gas with purity of 99.999% at a flow rate of 20 sccm. When 900 °C was reached, the RF power supply (GMPOWER PG-500) and RF matching network (GMPOWER BM-2000) were turned on. Furthermore, the pressure of the vacuum chamber was maintained at about 50 Pa after reaching the reaction setting time. The furnace was cooled down. To protect the as-synthesized GaN NWs from decomposition the flow of N_2_ was maintained during the cooling process. After cooling, a light-yellow layer was observed.

The structure, morphology and composition of the products were characterized using X-ray diffraction (XRD; BRUKER D8 ADVANCE), field emission scanning electron microscope (FESEM; JEM-2010F), and high resolution transmission electron microscope (HRTEM; Tecnai-F20). Raman spectroscopy was used to investigate the chemical-bond vibrational properties of the GaN samples, which were collected at room temperature using a T64000 micro-Raman spectroscopy. The photoluminescence (PL) of the specimen was measured at room temperature using a homemade photoluminescence spectroscopy, in which a 325 nm He-Cd laser was used as the excitation light source. Field emission properties were measured at room temperature in a vacuum ambient of 8.6  ×  10^−6^ Pa. ITO conductive glass and the as-synthesized nanowires were used as the anode and the cathode, respectively. The distance between them was 100 μm, and the emission current was monitored with a Keithley 2410 electrometer and recorded at a 1.0 interval by applying a sweep step of 5 V.

## Results and Discussion

### The effect of process parameters

The RF power and reaction time are the key factors for a PECVD system. During the reaction, it will significantly modulate the structures and performance of the as-synthesized nanowires. So the following two series of experiments were designed to study the specific mechanism of structural modulation. Detailed process parameters are shown in Table 1.

**Figure 2:**
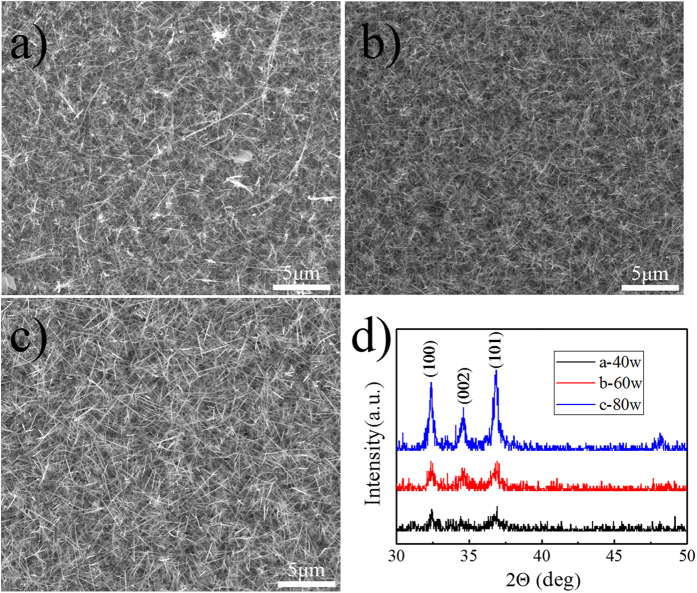
(**a**), (**b**) and (**c**) are the images of NWs synthesized on 40W, 60W, 80W, respectively. (**d**) XRD pattern of Series A.

As the XRD pattern in Fig. 2d shows, the crystallinity of these NWs was enhanced with the increase of RF powers, indicating a high tendency of crystalline growth after nucleation with a higher RF power. The analysis of FESEM and XRD pattern implies that the RF power may determine the crystalline quality of the nanowires.

**Figure 3:**
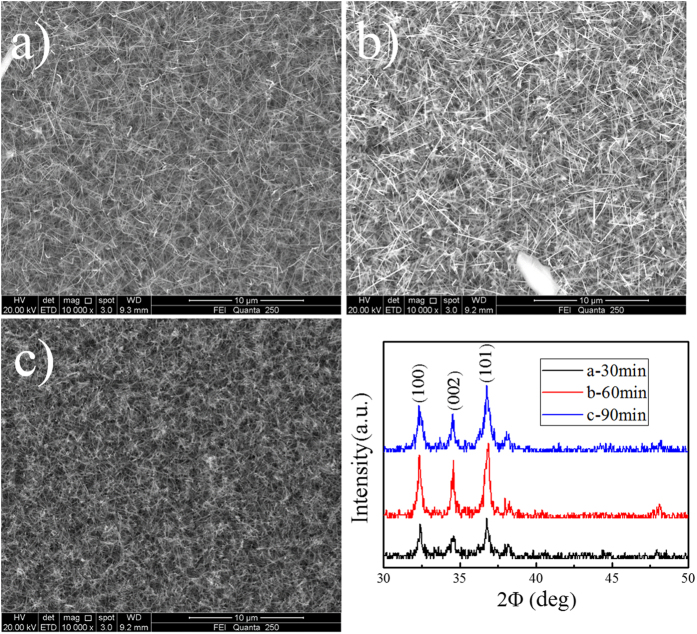
(**a**), (**b**) and (**c**) are the images of NWs synthesized on 30min, 60min, 90min, respectively. (**d**) XRD pattern of series B.

Figure 3(a) shows the morphology and spatial arrangement of the as-synthesized nanowires at the reaction time of 30 min. From the side view one can see that the nanowires were averagely slightly thinner than those at the other two reaction times (Fig. 3b,c). With the increase of reaction time the nanowires gradually grew thicker, while the length of them kept almost unchanged. This trend may be explained by the following argument: the precursor could radially diffuse deeper into the bulk of nanowires with a longer reaction time, so the wire diameter increased. However, the average wire length remained roughly the same due to the intense bombardment by high energy ions^9^. Figure 3(c) shows at the reaction time of 90 min. All nanowires were adhered together, thus intensifying the NWs agglomeration. The present study, in comparison with the results obtained by Brian A. Korgel^22^,is more in line with the following possibility: for a long time growth at a high temperature, the nanocrystals or liquid alloy droplets agglomerate, and then the nanowires become softened and stuck.

However, the change in XRD pattern (Fig. 3d) is not particularly obvious. It may suggest that the reaction time has no significant influence on the crystallization of nanowires. And the diffraction peak at ca. 38 degree was indexed to Au.

### The substrate effect

As well known, the substrate adopted often has a significant effect on the quality and structure of nanowires^23^. So, two experiments were designed as following (series C): NWs-a was synthesized on a Si wafer without any post-processing except cleaning; the substrate used for NWs-b was dipped in HF solution for 20 min.

**Figure 4:**
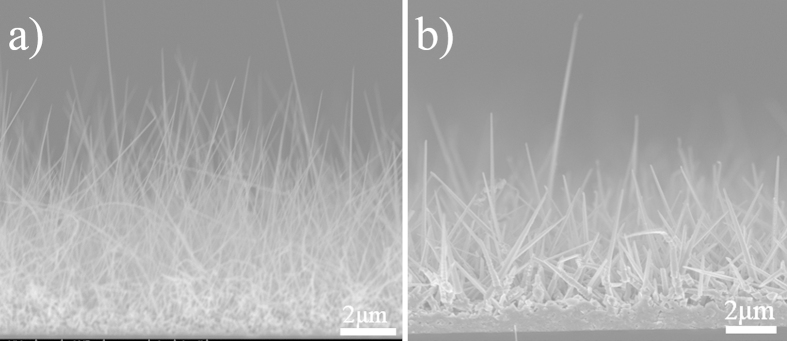
FESEM images of series C: (**a**) NWs-a grown on Si wafer without HF treatment, (**b**) NWs-b grown on Si wafer dipped in HF for 20min.

Figure 4 shows the FESEM images of the nanowires synthesized on the two different substrates. The RF power was 60 W and the other parameters as the same as series A (Table 1). Figure 4(a) shows the nanowires synthesized on a Si wafer without HF treatment. The as-prepared NWs are thin and a little kinked, with a typical diameter of less than 20 nm and the length in the range of 5 to 8 μm. From Fig. 4(b) one can see that by dipping in HF solution for 20 min the longest NW we can obtain has a length of ~7 μm and the length of most nanowires is between 1 and 3 μm. In addition, NWs all have a sharp tip.

**Figure 5:**
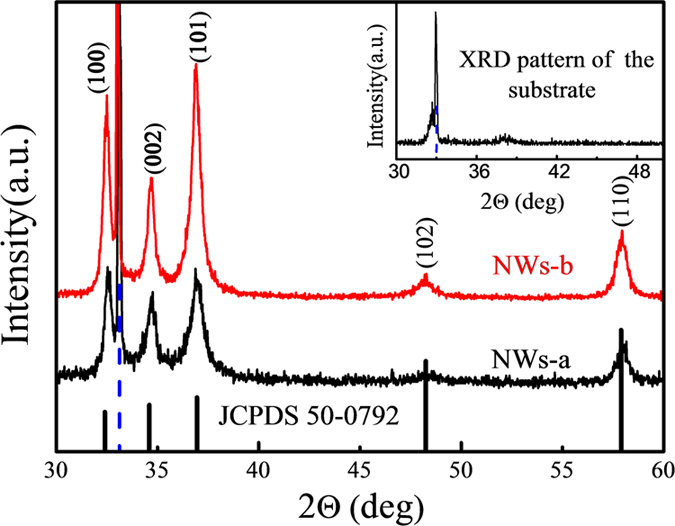
XRD pattern of series C. The insert is the XRD pattern of the substrate.

The overall crystal structure and phase purity of the nanowires were assessed through XRD measurements. As showed in Fig. 5, there are 6 peaks respectively at about 32.4°, 34.6°, 36.9°, 48.2° and 57.9°. This system can be indexed with respect to the wurtzite GaN structure (PDF# 50-0792). These sharp diffraction peaks suggest a good crystallinity. The dashed line highlighting the peak at about 33° is related to the diffraction peak of the Si substrate (as shown in the inset). From Fig. 5, it is obvious that the intensity of peaks in NWs-b is higher than that of NWs-a, which probably indicates that NWs-b have a better crystallinity or structural ordering.

**Figure 6:**
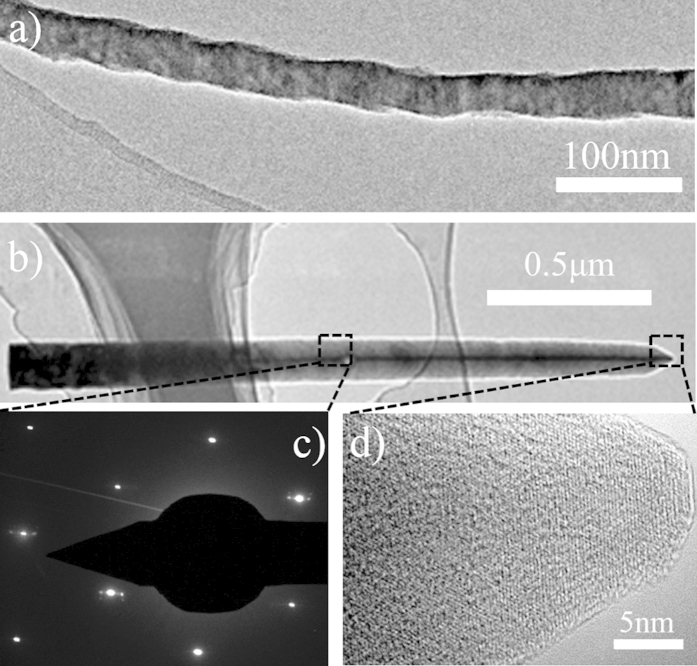
TEM images of series C: (**a**) NWs-a and (**b**) NWs-b. (**c**) SAED pattern and (**d**) corresponding HRTEM images of NWs-b, highlighted by the dashed boxes in (**b**).

TEM analyses were used to further characterize the structure of GaN NWs. As shown in Fig. 6(a), NWs-a prepared on the substrate without HF treatment exhibit a corrugated surface with a typical diameter of less than 20 nm. When the nanowires were deposited on the substrate dipped in HF solution for 20 min, nanowires with a triangular cross section were obtained (Fig. 6(b)). These nanowires are slightly shorter than NWs-a and have a typical diameter of about 80 nm.

Furthermore, from the selected area electron diffractions (SAED) and HETEM image (Fig. 6(c)), it can be found that the SAED diffraction spots are regular, that is, the selected GaN nanowire is mono-crystalline with a hexagonal structure.

**Figure 7:**
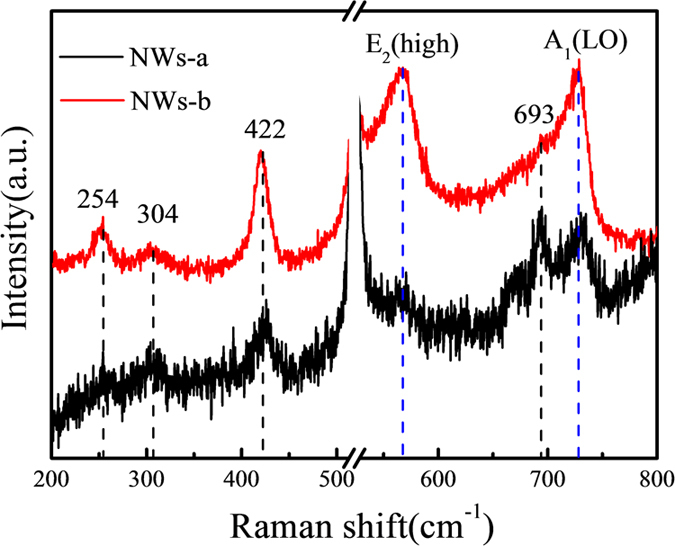
Raman spectra of series C.

Figure 7 shows the Raman spectra of the as-synthesized NWs. There is a break region from 513 cm^−1^ to 528 cm^−1^, where is the Si mode. And 304 cm^−1^ scattering line also belongs to Si mode^24^,^25^. The two peaks at about 568 cm^−1^ and 727 cm^−1^ are assigned to first-order phonons of E_2_ (high) and A_1_ (LO), respectively^26^,^27^,^28^.

From the spectra, one can obviously observe that the intensity of NWs-b is much higher than that of NWs-a. This means that NWs-b has a better quality, which is consistent with the analysis of Fig. 5. As for the A_1_ (LO) mode, GaN NWs display a red shift due to the small size confinement effect, as compared to the bulk material whose A_1_ (LO) locate at 736 cm^−1^.

Moreover, we also find three extra peaks at 254 cm^−1^and 422 cm^−1^, which can be attributed to the finite-size effect and high surface disorder degree^29^. There is also a peak that only can be observed in the spectra of NWs-a, at 693 cm^−1^. According to the study of Hsiang-Lin Liu^27^, the occurrence of this peak is due to the existence of defects in the as-synthesized NWs.

### Growth mechanism

**Figure 8:**
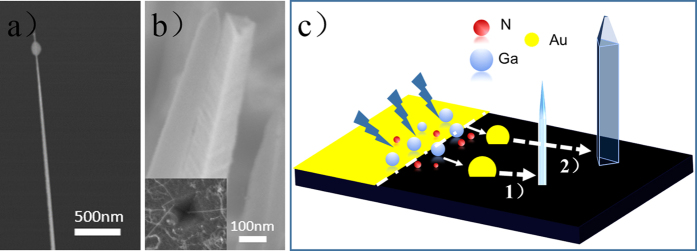
(**a**), (**b**) FESEM images of the two types nanowire and the surface treated by HF (Figure.8b insert), (**c**) Schematic of formation of GaN nanowire.

In the synthesis strategy, mainly affected by the substrate and RF power, GaN NWs with two different types of cross-sections were obtained. Just as shown in Fig. 8, the one looks like a needle with a small alloy droplet on the tip (Fig. 8(a)), which provides a crisp evidence that the growth of nanowires went through a vapor-liquid-solid (V-L-S) process^30^, while the other one has a triangular cross section (Fig. 8(b)). In this section, the growth mechanism of the two types of GaN NWs will be discussed in detail.

In this work, due to the difference of thermal expansion coefficients between gold and silicon wafer, the gold film will break into particles and melt into small droplets. Generally, Ga_2_O_3_ will react with carbon and decomposes into Ga_2_O, eventually reduced to metallic gallium^15^. The reactions can be expressed as


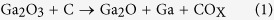



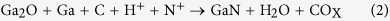


Furthermore, coupled with the effect of RF discharge, N_2_ will be ionized. Then, just as the standard VLS growth mechanism described^31^, the catalyst gradually forms catalyst-gallium-nitrogen alloys after the concentration of gallium nitride saturates. The droplet can act as a nucleation site, and a GaN nanowire begins to grow in one direction (Fig. 8(c)). In the foregoing process^32^,^33^, a high enough surface energy of liquid alloy in the droplet is needed to maintain the triple phase line (TPL, separating the vapor, liquid, and solid phases) stable on the top. However, in this work, due to the introduction of high-energy plasma the surface of the nanowire becomes hyperactive under the ion irradiation. Therefore, the downward force of surface energy 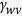 (NW sidewalls in contact with the vapor) will be much higher than the sum of upwards 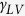 (the liquid-vapor interface) and 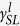 (the vertical solid-liquid interface). Thus, the TPL will move slightly downward. In other words the nanowire grows by wetting the sidewall of nanowires (Fig. 8(a)). Thus, more area will be wetted during this process, so the absorption of precursors will be accelerated and the nucleation will speed up. As compared to the catalyst-free spontaneous formation strategy^34^, we can achieve longer nanowires more easily by the similar process.

We stress the RF power as an important factor for preparing high-purity and -quality nanowires, which is consistent with the results obtained in the series A. Here, the effects of plasma for the growth of GaN NWs are emphasized. In the process of wetting, under the influence of high temperature and high-energy plasma, droplets will gradually decrease so the obtained nanowires all look like needles (Fig. 8(c)). As we all know, on the VLS growth the morphology of catalyst depends on the shape of nanowires. From the insert of Fig. 8(b), one can see that defects of inverted pyramid will form on the silicon wafer surface after corrosion by HF. This shape will limit the nucleation process that decides the ultimate nanowire morphology.

### Photoluminescence (PL) properties

**Figure 9:**
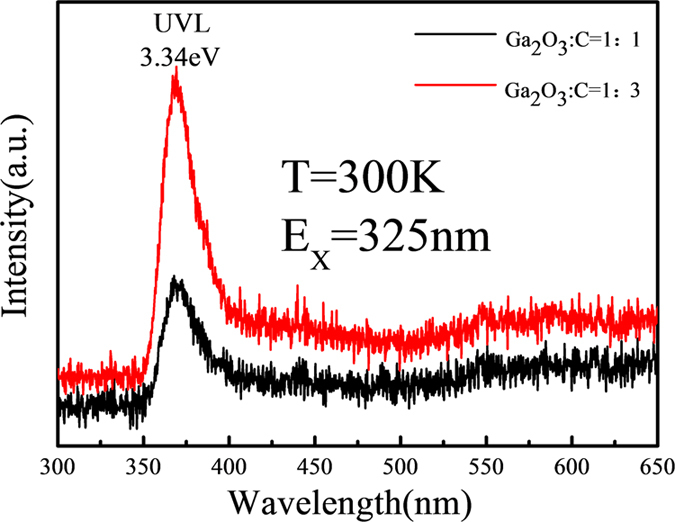
PL spectra of series C.

The PL spectra were obtained with a source of 325 nm excited by a He-Cd laser. It is known that pristine GaN will exhibit strong ultraviolet luminescence (UVL) at room temperature. From Fig. 9 one can see that there is a significant UVL band at about 3.34 eV, which indicates a good quality of the nanowires. Furthermore, when the molar ratio of Ga_2_O_3_ and C was 1:3 the UVL peak is much higher than that for the ratio of 1:1, which implies that increasing the incorporation amount of carbon powders can significantly improve the quality of the nanowires.

According to the previous studies by Reshchikov^35^, Kucheyev^36^ and Livneh^29^, there will be a yellow luminescence due to the C impurities and other defects. However, there is no other luminescence peaks in the spectra. It may indicate that the carbon composition in the reaction has been well controlled, and the quality of the nanowires can be significantly adjusted by controlling the amount of carbon.

### Field emission (FE) properties

**Figure 10:**
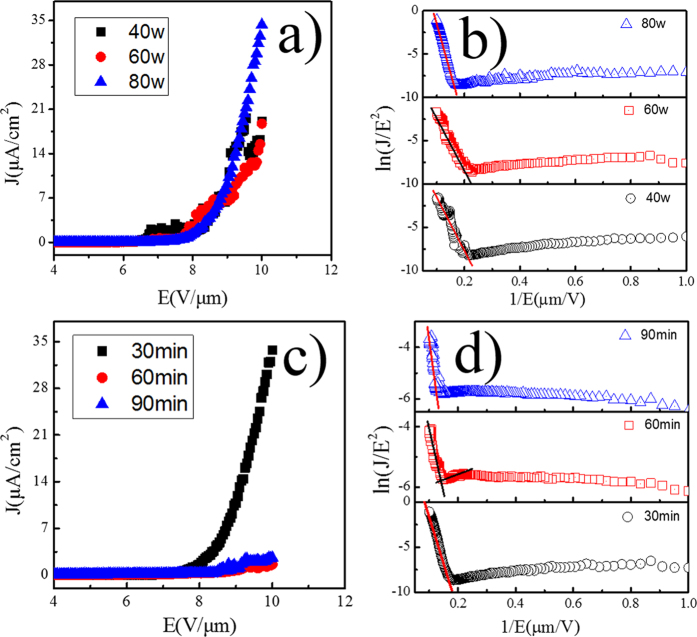
FE characteristics of the series A and series B: (**a**) (**c**) emission current density versus applied electricfield (J-E) curve; (**b**) (**d**) F-N plot.

Figure 10 shows the FE current density versus applied field (J−E) characteristic and Fowler−Nordheim (F−N) curves of Series A and series B, respectively. All two groups of experimental data are shown. As the applied voltage is gradually increased, the emission current is observed to increase very rapidly, indicating that the electron emission in accords with F–N tunneling. In this paper, we define the turn-on field as the electric field required to produce an emission current density of 1 μA·cm^−2^.

Figure 10(a) shows the field emission plots for the GaN NWs prepared with different RF powers. The turn-on fields of NWs synthesized at 40 W, 60 W, and 80 W are about 6.8 V μm^−1^, 7.8 V μm^−1^ and 8 V μm^−1^, respectively. This indicates that all of them have a similar turn-on field. However, in the condition of 10 V μm^−1^ the maximum current density of NWs synthesized at 80 W is about ~35 μA cm^-2^, significantly larger than the other two. As we all know, the nanowires with top-tips along specific orientations can greatly enhance field electron emission because of a strong local electric field at their tips. The previous analysis showed that when the RF power was up to 80 W, the proper structure of the prepared nanowires was more helpful to improve the maximum field emission current density.

Figure 10(c) shows the J–E with F-–N curves of series B. From Fig. 10(c) one can see that the turn-on fields of NWs synthesized at 30 min, 60 min, and 90 min are about 7.8 V μm^−1^, 9.2 V μm^−1^ and 9 V μm^−1^, respectively. Furthermore, the maximum current density of NWs synthesized at 30 min is nearly 10 times larger than the others. Just as Fig. 3(a) shows, the nanowires synthesized in the condition of 30 min have a larger surface area and length-diameter ratio, which can advance the field emission maximum current density^37^,^38^.

In addition, the FE property of a flat multi-tip emitter can be analyzed by F–N equation^39^:


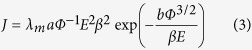


Where J is the emission current density, a and b are F–N constants, E is the average applied electric field, Φ is the work function, β is the field enhancement factor, 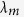
is a macroscopic pre-exponential correction factor. From the previous studies by Suryawanshi^39^, for a multi-tip emitter the electric field can be written as E_local_ = β E_average_, so the field enhancement factor β will be overestimated. Hence, we did not estimate the value of β in our present study.

In Fig. 10(b), we can see that all the curves of the two series can be divided into two linear parts. The linear part indicates that the electron field emission properties of all the samples are close to the F–N emission mechanism. However, the second part of the curves deviates from F–N plot. According to equation (1), we conclude that it may be originated from the effect of morphology damage and the absorbents for NWs. During FE testing process the generated Joule heat will damage the morphology of GaN NWs, so a number of nanowires were flattened and shortened. The effective emission area will be reduced, which will lead to a drift of the F–N line.

These results suggest that nanowires with a large diameter, surface area and length diameter ratio have a better field emission property. However, the generated Joule heat and adsorption gas are the barriers for stable electron emission. Simultaneously, these results enrich our knowledge on improvement of the field emission properties and are helpful for us to understand the mechanism of nanowires electron emission.

## Conclusion

By an improved PECVD method, all materials used in this technique are Ga_2_O_3_ and carbon powders, N_2_ and H_2_, which are all cheap and easily to acquire. GaN nanowires were prepared by a simple and green strategy. The high crystalline NWs with a length of 20 μm and maximum diameter of 143 nm were obtained. The influences of substrate, RF power and reaction time on the growth of GaN nanowires were systematically studied. The results suggest that the defects on the substrate formed by dipping in HF will contribute to nucleation and growth of nanowires. The RF power is the key to prepare GaN NWs. It has an obvious effect on the nucleation and growth process. The results reveal that a 80 W of RF power is optimum for the growth of nanowire in the PECVD system. The reaction time can also significantly affect the morphology, but when the reaction time reaches to 90 min nanowires will stick together. Furthermore, the possible growth mechanism was also discussed. The PL and FE spectra showed that the adjustment of process parameters can significantly influence their performance. This work supplies a low cost and green method to synthesize GaN NWs, providing more choices of equipment and materials for preparing GaN low dimensional materials. The method will help us to understand the regulation mechanism for preparing high quality nanowires in a high temperature and plasma environment.

## Additional Information

**How to cite this article**: Zhao, J.-W. *et al.* A low cost, green method to synthesize GaN nanowires. *Sci. Rep.* **5**, 17692; doi: 10.1038/srep17692 (2015).

## Figures and Tables

**Table 1:** Detailed growth conditions including RF power, catalyst (Au), flow rate of gas, reaction temperature (*T*) and growth time (*t*).

series	specimen	RF power(W)	t (min)	Au (s)	H_2_ (sccm)	N_2_ (sccm)	T (°C)
A	a	40					
	b	60	60	10	10	20	900
	c	80					
B	a		30				
	b	80	60	10	10	20	900
	c		90				
